# Personalized non-invasive neuromodulation for sensory-based urge suppression in individuals with OCD: a proof-of-concept investigation

**DOI:** 10.3389/fnhum.2025.1587644

**Published:** 2025-06-24

**Authors:** Goi Khia Eng, Arielle Tambini, Molly S. Hermiller, Nicolette Recchia, Jeanmarie R. Harvey, Dan V. Iosifescu, Russell H. Tobe, Emily R. Stern

**Affiliations:** ^1^Department of Psychiatry, New York University Grossman School of Medicine, New York, NY, United States; ^2^Clinical Research, Nathan S. Kline Institute for Psychiatric Research, Orangeburg, NY, United States; ^3^Department of Psychology, Florida State University, Tallahassee, FL, United States; ^4^Center for the Developing Brain, Child Mind Institute, New York, NY, United States; ^5^New York University Grossman School of Medicine, Neuroscience Institute, New York, NY, United States

**Keywords:** transcranial magnetic stimulation, obsessive-compulsive disorder, sensory phenomena, sensory urges, urges-for-action, fMRI, individualized, postcentral gyrus

## Abstract

Obsessive-compulsive disorder (OCD) is chronic and impairing. While OCD often involves fear of harm or bad events, many patients experience “sensory phenomena,” which are aversive sensory experiences that drive repetitive behaviors regardless of specific fears. Standard treatments do not effectively address sensory phenomena, and novel approaches are needed. Transcranial magnetic stimulation (TMS) is a safe and non-invasive neuromodulation technique increasingly used in psychiatric disorders, including OCD. This work presents a data-driven approach to identifying TMS brain targets for modulating sensory urges in OCD incorporating both behavioral and clinical criteria (Study 1) for a proof-of-concept investigation (Study 2). Study 1 included 69 individuals with OCD and 23 controls who completed an urges-for-action fMRI task involving instructed eyeblink suppression as an experimental model for sensory-based urges. Data-driven conjunction analysis revealed several brain regions, including the right postcentral gyrus, that were associated with more blink suppression failure (behavioral), more severe sensory phenomena (clinical), and were hyperactivated in OCD patients compared to controls. Study 2 administered single-session inhibitory TMS on 4 returning OCD patients using individualized targets within the postcentral gyrus identified from Study 1. Compared to sham, inhibitory TMS delivered to individualized postcentral gyrus targets resulted in fewer blink suppression failures, reduced activation in the target (postcentral gyrus) and key urge-related areas (insula, mid-cingulate), and greater reduction in self-reported urge to engage in OCD-related compulsions, with medium to large effect sizes. These findings demonstrate the potential of utilizing data-driven approaches incorporating behavioral and clinical criteria to target hard-to-treat sensory phenomena in OCD.

## 1 Introduction

Obsessive-compulsive disorder (OCD) is a chronic and debilitating disorder that affects 2 to 3% of the global population (Ruscio et al., 2010; Kessler et al., 2012). OCD is characterized by the presence of obsessions (i.e., recurrent, intrusive urges or thoughts that cause distress) and/or compulsions (i.e., time-consuming and repetitive behaviors or mental rituals that are performed to reduce anxiety caused by obsessions). Although well-known symptoms of OCD involve fear of harm or bad events (Frost and Steketee, 2002; Abramowitz et al., 2009), as many as 65% of individuals with OCD also report “sensory phenomena,” which are aversive sensory experiences such as physical sensations and sensory-based urges that are similar to premonitory urges prior to tics (Kurlan et al., 1989; Cohen and Leckman, 1992; Leckman et al., 1993; Kwak et al., 2003; Shavitt et al., 2014). Unlike harm- and fear-based OCD symptoms, where the purpose of compulsions is to reduce anxiety and/or prevent a dreaded event (e.g., checking the stove to prevent fire), these “tic-like compulsions” are performed to reduce an uncomfortable urge and/or to achieve a “just-right” sensation (Rosario et al., 2009; Ferrão et al., 2012; Shavitt et al., 2014; Katz et al., 2022).

Prior work has suggested that sensory-based urges in OCD are phenomenologically similar to everyday “urges-for-action” (UFA), which are normative urges to perform a behavior, such as the urge to blink, cough, or scratch (Jackson et al., 2011; Berman et al., 2012; Leech et al., 2013; Mazzone et al., 2013; Botteron et al., 2019). Like sensory-based urges, UFAs intensify the longer they are suppressed and temporarily subside when acted upon (Miguel et al., 2000; Berman et al., 2012; Brandt et al., 2018). UFAs activate an “urge network” consisting of sensorimotor-related brain regions including the postcentral gyrus, insula, and mid-cingulate cortex (Jackson et al., 2011), areas which have also been associated with sensory phenomena symptoms in OCD (Subirà et al., 2015; Brown et al., 2019).

Leveraging this similarity, our research has utilized a UFA fMRI paradigm to elicit the urge to blink via instructed blink suppression as an experimental model for sensory-based urges in OCD (Stern et al., 2020; Bragdon et al., 2023; Eng et al., 2024). This approach enables the characterization of urge-related behavior and brain function in OCD without relying on self-report or evoking idiosyncratic symptoms. Additionally, it allows for the direct comparison of sensory urge-related activity between OCD and control participants as the paradigm elicits the urge to blink in the vast majority of individuals (Berman et al., 2012; Stern et al., 2020). Using this task, we previously reported that OCD patients exhibited more failures of urge suppression (i.e., they made more erroneous blinks when instructed to withhold blinking for an extended duration) (Stern et al., 2020; Bragdon et al., 2023) and had greater activation in key urge network regions, including the postcentral gyrus, insula, and mid-cingulate compared to controls (Stern et al., 2020). In a machine learning analysis in the same cohort, we identified three distinct subgroups of OCD patients based on the number of blink suppression failures on the UFA fMRI task. The OCD subgroup with the highest number of blink suppression failures (i.e., highest number of erroneous blinks) had the most severe sensory phenomena symptoms and highest activation in urge network regions compared to the other subgroups, despite there being no differences in overall OCD severity among subgroups (Eng et al., 2024). These investigations highlighted the effectiveness of the UFA fMRI paradigm in eliciting sensory-based urges and neural activity in urge network regions and validate the use of the task as an experimental model of sensory-based urges in OCD.

Despite their prevalence and clinical significance in OCD, sensory phenomena are not well addressed by standard treatments such as behavior therapies and pharmacological approaches involving serotonin reuptake inhibitors. Behavior therapies that target fears and cognitions are not as readily applicable to the treatment of sensory phenomena. Similarly, sensory phenomena may be less responsive to serotonin reuptake inhibitors monotherapy than harm-related symptoms (Foa et al., 1999; Abramowitz et al., 2003; Mansueto and Keuler, 2005; Stein et al., 2007). Even in cases where sensory phenomena do respond to these first-line treatments, the overall low remission rates (~50%) suggest the need for more targeted approaches (Davidson and Bjorgvinsson, 2003; Simpson et al., 2006; Springer et al., 2018).

Transcranial magnetic stimulation (TMS) is a non-invasive neuromodulation technique that has been used to treat a variety of conditions including OCD (Cohen et al., 2022). TMS involves placing a magnetic coil over the head to generate strong magnetic fields that induce brief electric currents in the brain area under the coil. TMS can be used to produce excitatory or inhibitory effects on brain tissue depending on the stimulation parameters (Hallett, 2007; Brunoni et al., 2019). One TMS protocol is FDA-approved for the treatment of OCD, which applies high-frequency excitatory TMS to the medial prefrontal/anterior cingulate cortices (mPFC/ACC) (Carmi et al., 2019). While the initial efficacy study was promising, subsequent attempts to ameliorate OCD symptoms using the same protocol yielded poor effect sizes, and it had low uptake in specialized OCD clinics (Grassi et al., 2023). Clinical trials using TMS in OCD have also tested other targets, including dorsolateral prefrontal cortex, orbitofrontal cortex, and supplementary motor area (Grassi et al., 2023; Kar et al., 2024). Although these targets have shown some promise in reducing overall OCD severity, findings have been highly variable (Grassi et al., 2023; Vinod et al., 2024), which may be due in part to symptom and neural variability in the cohorts being tested (Grassi et al., 2023). Few TMS studies have targeted specific symptoms or phenotypes in OCD, and to our knowledge, none has focused specifically on sensory-based urges in OCD.

The current manuscript describes results from a pilot study testing the effects of TMS on sensory-based urges in OCD. Study 1 employed a data-driven method for target identification using clinical and behavioral measures of sensory-based urges, identifying a region in the right postcentral gyrus as a TMS target. Study 2 was a proof-of-concept investigation comparing the effects of single-session individualized TMS targeting this postcentral gyrus region versus sham on behavior and brain activation in the UFA fMRI task in a pilot sample of four patients with OCD.

## 2 Study 1: data-driven identification of TMS target region using clinical and behavioral measures of sensory-based urges

### 2.1 Method

#### 2.1.1 Participants and procedure

Participants were recruited and scanned at the Icahn School of Medicine at Mount Sinai (ISMMS), Nathan Kline Institute for Psychiatric Research (NKI), and New York University Grossman School of Medicine (NYUSoM) between May 2017 and September 2020 (Supplement 1.1). The analyzed sample included 69 individuals with OCD and 23 control participants who had useable neuroimaging and eyeblink data (Supplement 1.2–1.3). The participants included in this dataset were part of a larger set of neuroimaging studies, data from which have been published previously (Stern et al., 2020; Bragdon et al., 2023; Eng et al., 2024). All subjects provided written informed consent.

Clinical interviews assessed for current DSM-5 diagnoses [Mini International Neuropsychiatric Interview; M.I.N.I (Sheehan et al., 1998)] and rated overall OCD severity [Yale-Brown Obsessive Compulsive Scale; Y-BOCS (Goodman et al., 1989)] as well as severity of sensory phenomena specifically [University of Sao Paolo’s Sensory Phenomena Scale; USP-SPS (Rosario et al., 2009; Sampaio et al., 2014); Supplement 1.4]. Information on comorbid conditions and the use of psychotropic medications is in the Supplement 1.5–1.6.

#### 2.1.2 UFA fMRI task

This task elicits sensory-based urges by asking participants to suppress eyeblinks for prolonged periods of time [also see Stern et al. (2020)]. Participants were asked to withhold blinking for a period of 60 s (indicated by the word “HOLD” in the center of the screen). After 60 s had elapsed, participants were permitted to blink (“OK TO BLINK” recovery period, 4 s) and then rated the subjective strength or intensity of the urge experienced during the prior suppression period on a 5-point scale (1 = “Not strong at all/no urge” to 5 = “Extremely strong”; up to 4 s). Blocks of blink suppression alternated with 30-s blocks of free blinking (“NORMAL”). Inter-stimulus and inter-trial intervals were jittered between 2 to 5 s, with leftover time from the urge rating (if made before the full 4 s had elapsed) added to the inter-trial interval, to reduce event collinearity. Participants were instructed to immediately resume withholding blinking should any “erroneous” blinks occur during the suppression period. Eyeblinks were measured via pupil occlusion using Eyelink 1000-Plus (SR Research, 2016) during the fMRI task. A total of eight blocks of blink suppression and eight blocks of free blinking were presented over two runs, for a total task length of approximately 15 min.

### 2.2 Data analysis

Information on structural and functional neuroimaging data acquisition are detailed in the Supplement 1.7.

Using the UFA fMRI task, we previously reported greater activation in OCD patients compared to controls in multiple urge network regions including the postcentral gyrus, insula, and mid cingulate during the initial 30 s of the 60 s-suppression period as the urge begins and starts to build (Stern et al., 2020). By contrast, fewer areas of hyperactivation were found during the later phases of urge suppression, where both groups exhibited robust activation of urge network regions (Stern et al., 2020). These findings suggest that individuals with OCD may experience urges differently than controls particularly during the early part of blink suppression. Therefore, analyses for the present study focused on the early phase of the suppression period (first 30 s).

Erroneous blinks committed during suppression blocks were averaged across all eight suppression blocks and then square-root transformed due to skewness [(Bragdon et al., 2023) see Supplement 1.3 for data cleaning procedures], with a greater value reflecting more “impairment” or failures of urge suppression in the task. Ratings of urge intensity following suppression periods were averaged across all blocks.

#### 2.2.1 Neuroimaging data preprocessing and analyses

Preprocessing was performed using a combination of Statistical Parametric Mapping (SPM) v.12, AFNI v.10.6, and FSL v.5.0.10 (Supplement 1.7.1). Following preprocessing, a fixed-effects general linear model was created at the individual subject level to model the BOLD signal separately during the early phase of suppression (i.e., hereafter “suppression”; first 30 s of the 60 s-suppression period), late phase of suppression (last 30 s), and free-blinking with block regressors (Supplement 1.7.1).

Three random-effects models were created to probe brain areas related to impairment of urge suppression and clinical symptoms of sensory phenomena in the OCD sample. Whole-brain group-level regression analyses examined the relationship between greater neural activation during suppression and: (1) more failures of blink suppression and (2) higher severity of sensory phenomena (i.e., higher scores on the USP-SPS). A third model compared neural activation between a sub-sample of OCD patients with more pronounced impairment of blink suppression (i.e., patients with erroneous blinks exceeding the median value of the OCD group) (*n* = 37) and control participants using two-sample *t*-tests. MRI scanning site was specified as a covariate-of-no-interest in all analyses. In order to identify a common set of neural regions both clinically and behaviorally relevant for urge suppression in OCD, a conjunction analysis was conducted across all three group-level analyses, revealing common neural regions associated with the clinical severity of sensory phenomena within OCD (#2), and impairments in urge suppression during the experimental UFA task both within OCD (#1) and between OCD and controls (#3) (*p* < 0.005, uncorrected for each whole-brain analysis).

### 2.3 Results

Supplementary Table 1 presents demographic, behavioral, and clinical characteristics for OCD patients and controls (also see Supplement 1.8). Within the OCD group, patients with more failures of blink suppression also reported greater intensity of the urge to blink (*r* = 0.34, *p* = 0.004), and had more severe clinical symptoms of sensory phenomena (*r* = 0.28, *p* = 0.018). Sample characteristics for the OCD sub-sample who had erroneous blink count greater than the median of the full OCD group (*n* = 37) are presented in Supplementary Table 2.

**Figure 1:**
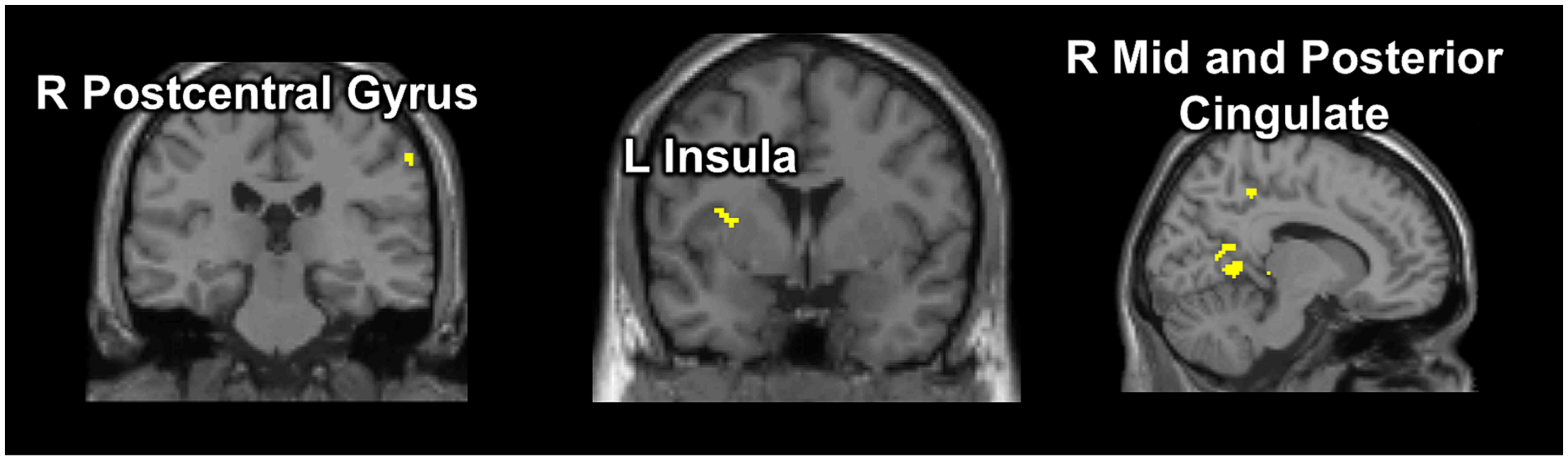
Selected results from the conjunction analysis. Our data-driven approach using conjunction analysis identified a common set of brain regions activated during the early phase of suppression in the UFA fMRI task that were associated with behavioral performance (greater number of erroneous blinks during suppression) and clinical severity (sensory phenomena score) within the patient group (OCD *n* = 69), and were also more activated in OCD patients exhibiting more failures of blink suppression (OCD_Sub-sample_ *n* = 37) than control participants (*n* = 23). *p* < 0.005 (uncorrected).

The group-level fMRI conjunction analysis identified several brain areas, including postcentral gyrus, precuneus, mid and posterior cingulate, insula, caudate and occipital cortex (superior occipital, calcarine, and lingual gyrus) (Figure 1).

#### 2.3.1 Postcentral gyrus as TMS stimulation region

**Figure 2:**
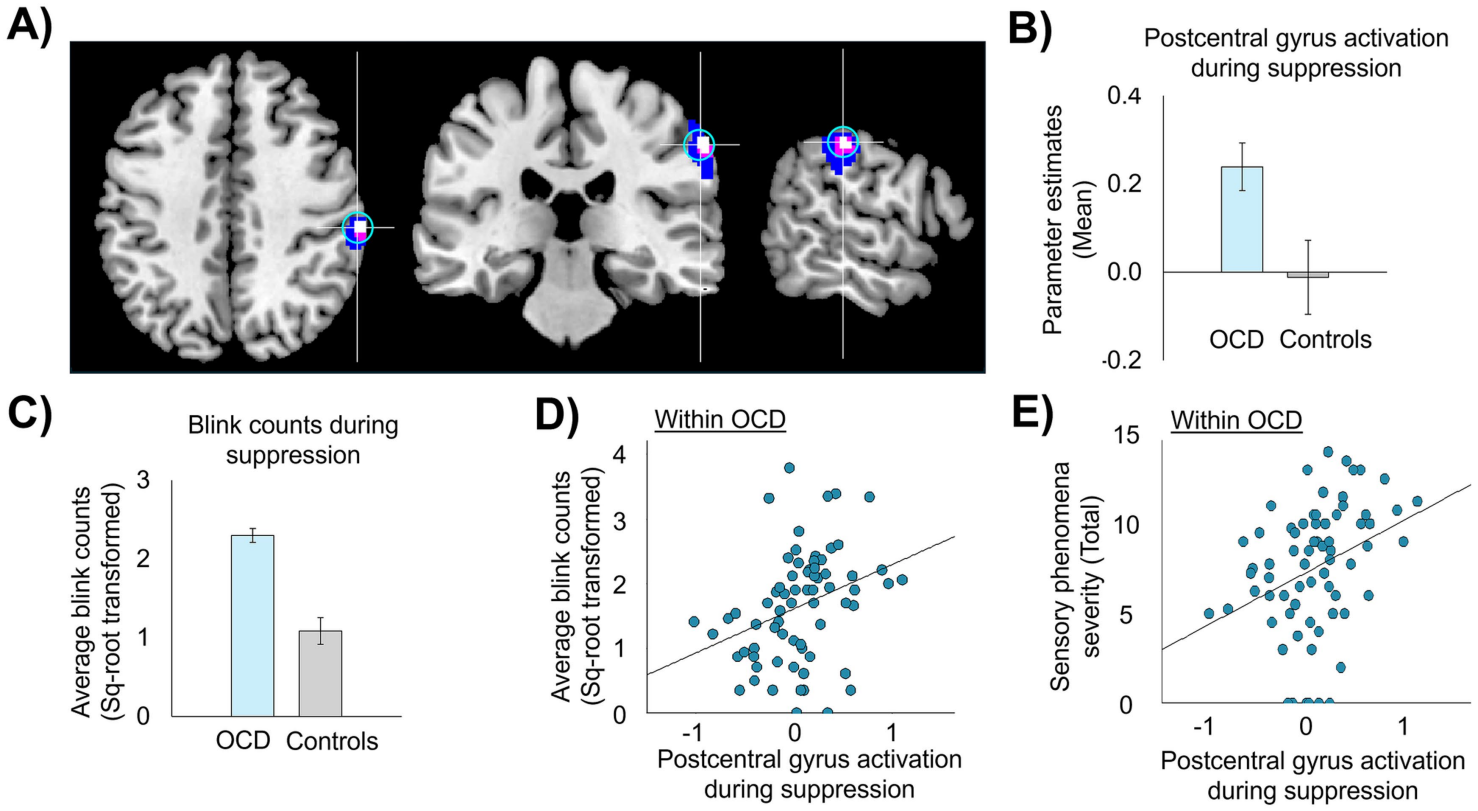
Right postcentral gyrus activation during suppression and association with failures of blink suppression failure and sensory phenomena. **(A)** Image of the right postcentral gyrus stimulation region (MNI: 60, −26, 42) derived from conjunction analysis, *p*-uncorrected <0.005 (in white), with spread of activation at lower thresholds (*p* < 0.01 in violet and *p* < 0.05 in blue); a 7 mm-radius sphere (in cyan) centered on the postcentral gyrus coordinate. **(B,C)** Group differences between controls (*n* = 23) and a sub-sample of OCD patients (*n* = 37) with higher suppression failures (>median of the patient group) in activation in the postcentral gyrus sphere **(B)** and erroneous blink counts during suppression **(C)**. **(D,E)** Scatterplots showing associations between activation in postcentral gyrus with failures of urge suppression **(D)** and severity of sensory phenomena **(E)** within the full sample of OCD patients (*n* = 69). Statistical analyses were not performed as doing so would be circular, given that the right postcentral gyrus coordinate was derived from conjunction analyses performed on these relationships.

Out of the regions identified with the conjunction analysis, the postcentral gyrus (MNI: 60, −26, 42) was selected as the target for TMS stimulation region (Figure 2) for the following reasons: first, the identified postcentral gyrus area is located closer to the surface of the brain and is more easily reached with the butterfly TMS coil (figure-eight coil) used in this study (MagVenture Cool-B65 A/P coil) (Deng et al., 2013) than the other regions identified. Second, the postcentral gyrus is consistently associated with the detection of sensation, including encoding the intensity of a sensory experience (Leech et al., 2013; Güçlü et al., 2015; Gu et al., 2024; Zouki et al., 2024), and is part of the urge network that activates in response to different types of urges-for-action in controls and patient populations (Simmons et al., 2013; Satpute et al., 2015; Zouki et al., 2024). This evidence supports the selection of the postcentral gyrus as an appropriate target for modulating sensory-based urges in OCD. Figure 2 displays group differences in postcentral gyrus activation (Figure 2B) and blink counts (Figure 2C) during suppression as well as the associations between task-related postcentral gyrus activation with blink counts (Figure 2D) and severity of sensory phenomena (Figure 2E) within the OCD sample. Note that data in Figure 2 were presented for display purposes only and were not statistically analyzed in order to avoid circularity.

## 3 Study 2: proof-of-concept neuromodulation pilot study targeting the postcentral gyrus

**Figure 3:**
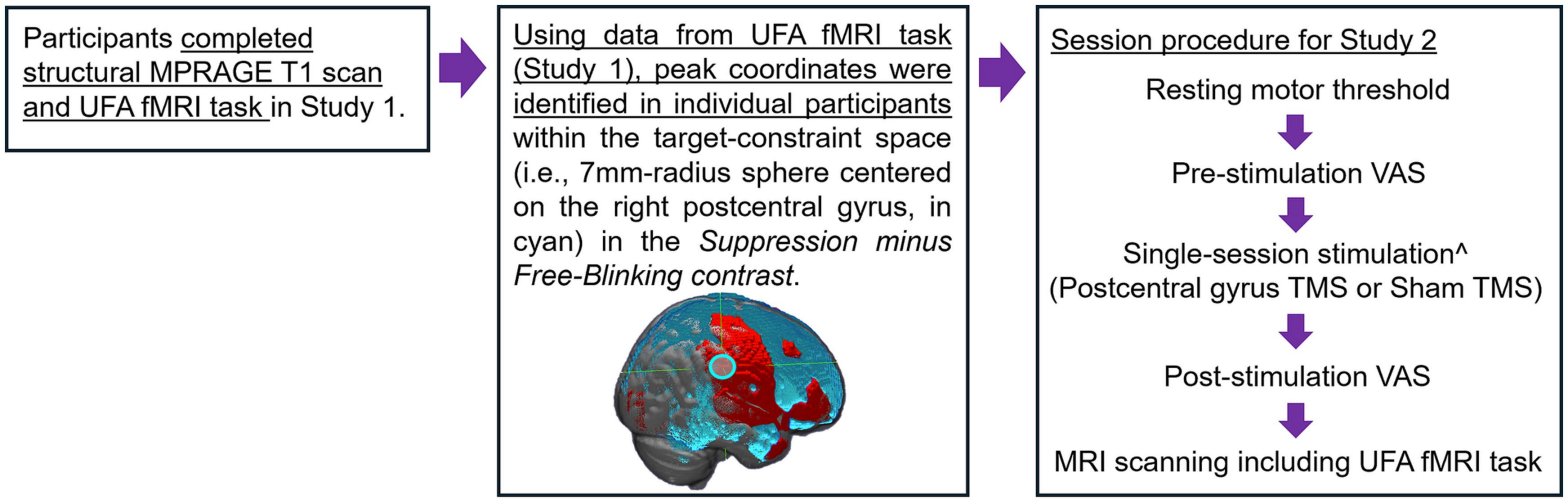
Procedural figure for Study 2. ^Single-session stimulation with neuro-navigation. Order of stimulation condition was counterbalanced, and stimulation sessions were separated by at least 5 days.

Study 2 examined the effects of inhibitory TMS targeting the postcentral gyrus region identified from Study 1 on behavioral and neural markers of urge suppression in a small pilot sample of OCD patients (Figure 3). It was hypothesized that, compared to sham stimulation, active inhibitory TMS would improve patients’ ability to suppress sensory-based urges (i.e., reduce failures of blink suppression in the task) and reduce activation not only in the postcentral gyrus target region but also in other key urge network regions such as the insula and mid-cingulate.

### 3.1 Method

#### 3.1.1 Participants

Four patients with OCD who completed Study 1 were brought back into the lab between July and November of 2022 for our pilot TMS investigation (Supplement 2). All participants had previously completed a structural MPRAGE T1 scan and the UFA fMRI task in the scanner as a part of their participation in Study 1.

#### 3.1.2 Individualized target selection

Individual participant’s fMRI task data from Study 1 was used to provide a pre-TMS “baseline” measurement of suppression-related activation. The contrast *suppression minus free-blinking* was evaluated at the individual subject level with the TMS target defined as the coordinate with the highest T-value within the postcentral gyrus region-of-interest identified in the conjunction analysis [7-mm sphere around 60, −26, 42 in MNI space; Figure 3 (middle)]. Using SimNIBS, the postcentral gyrus coordinate information is transformed from MNI to native subject space using the *mni2subject coords* function. Subsequently, e-field modeling and simulation were conducted to determine the optimal TMS coil orientation and positioning to maximize the e-field induced at the target region (Saturnino et al., 2019; Padberg et al., 2021). Targeting information was imported into Localite TMS Navigation software to aid in neuronavigation real-time.

#### 3.1.3 Stimulation schedule and procedure

Participants received two sessions of TMS: one active session targeting the postcentral gyrus and one sham stimulation session. Sessions occurred on two different days in counterbalanced order, separated by at least 5 days to prevent cross-over effects, consistent with prior work (Singh et al., 2019; Villafuerte et al., 2019). To maintain blinding, participants underwent the same procedures and received the same set of instructions on both active and sham sessions. On both stimulation sessions, immediately before and after stimulation, participants filled out visual analogue scales (VAS) that asked them to rate the in-the-moment strength of their urge to perform their OCD-related compulsions (Figure 3). Ratings on the VAS were made by marking a point along a continuous line, with “not at all” and “extreme” on the left and right anchors, respectively. Immediately after completing post-stimulation VAS ratings, participants proceeded to the MRI scanner (located in an area directly adjacent to the stimulation room), where they performed the UFA fMRI task. Task fMRI scanning commenced within an average of 6 min 38 s (S.D. = 47 s) after the end of the stimulation (including post-stimulation VAS ratings). Prior work indicates that the inhibitory TMS protocol used here (see details below) causes neural effects lasting approximately 30–45 min (Goldsworthy et al., 2012; Wischnewski and Schutter, 2015; Strzalkowski et al., 2019). As such, the UFA task fMRI scanning (task duration ~15 min) is well within this timeframe, including the time needed to move participants into the scanner following stimulation. At the end of the visit, participants filled out a debriefing form asking whether they believed they had received real (i.e., active) or fake (i.e., sham) stimulation.

#### 3.1.4 TMS parameters

TMS was delivered using continuous theta-burst stimulation (cTBS), which is shown to induce cortical inhibitory effects (Cárdenas-Morales et al., 2010), using MagPro X100 Stimulator with MagOption and MagVenture Cool-B65 A/P figure-of-eight coil with frameless stereotactic neuronavigation (Localite TMS Neuronavigation). At the beginning of each session, resting motor threshold (RMT) was administered using MagVenture-B60 coil to establish the stimulation intensity for cTBS, determined as the lowest intensity that was required to reliably elicit a twitch in the contralateral thumb in 50% of single TMS pulses (Bystritsky et al., 2008; Marder et al., 2022). cTBS was administered as a burst of three biphasic waveform pulses at 30 Hz, repeated at 6 Hz for a total of 200 bursts (600 pulses) at 80% RMT (Goldsworthy et al., 2012).

To blind participants to the stimulation condition, sham stimulation was administered using the placebo side of the MagVenture Cool-B65 A/P coil, which does not deliver active stimulation but produces similar auditory clicking sounds. To mimic the tactile sensation of actual stimulation, in addition to the auditory clicking sounds from the coil, rubber electrodes delivering weak electrical current were attached to the participant’s scalp under the coil (Mennemeier et al., 2009). The left inferior parietal region (MNI: −48, −48, 50), which is contralateral and posterior to the active target, was selected as the target for sham stimulation. This study intentionally selected a sham target that was distant from the active target. This minimized participants directly comparing sensations between stimulation sessions, such as head or facial muscle twitches, which are not mimicked by the A/P coil and electrodes and could compromise blinding. Notably, for consistency in study procedures and to maintain blinding, rubber electrodes were also attached to the participant’s scalp during active stimulation but were not turned on to avoid pain.

### 3.2 Data analysis

Eyeblink and neuroimaging data were analyzed according to the procedures described in Study 1. Effects of TMS were examined by comparing the number of blink suppression failures and neural activation during suppression following active TMS compared to sham stimulation, as well as pre-to-post stimulation changes in the self-reported urge to engage in OCD-related compulsions (VAS ratings) recorded during each session.

Postcentral gyrus activation was analyzed by extracting parameter estimates from the blink suppression vs. free blinking task condition from within spheres surrounding individualized postcentral gyrus coordinates (see 3.1.2 Individualized target selection section above). Additionally, bilateral ROI masks of the insula and mid-cingulate cortices were created, and parameter estimates were extracted to examine neural responses to TMS in key regions of the urge network (in addition to the postcentral gyrus).

VAS ratings were converted to a percentage by measuring the distance of the mark made by the participant from the left anchor (zero) and divided by the total scale length.

Due to the small sample size for this pilot work, the results presented showed estimates of effect size with small sample size bias correction (Hedge’s *g*) rather than *p*-values. Effect sizes were computed using the *effsize* package (version 0.8.1) in R.

### 3.3 Results

**Figure 4:**
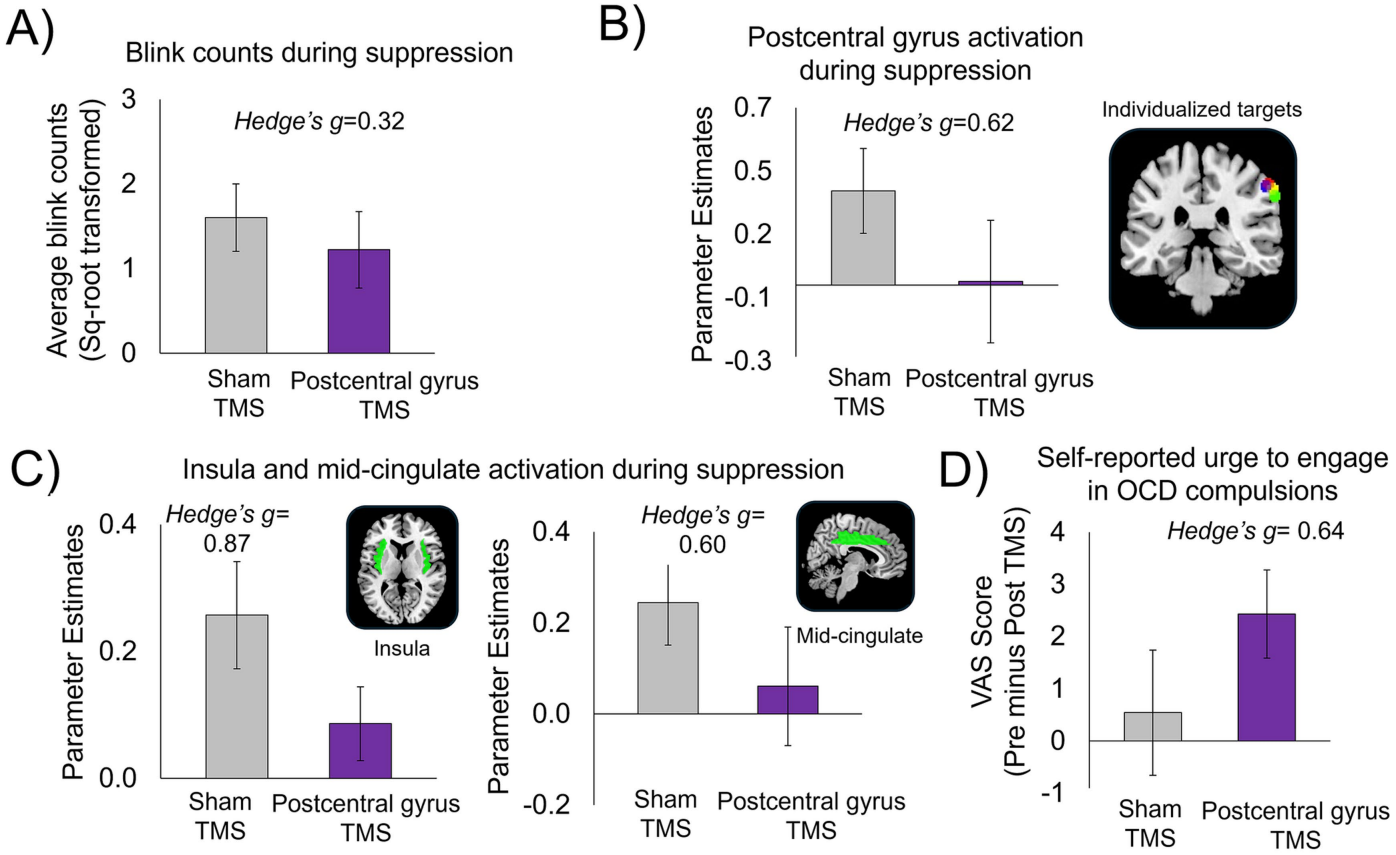
Results from the pilot proof-of-concept investigation, *n* = 4 patients with OCD. Following inhibitory TMS to the postcentral gyrus compared to sham TMS, patients showed: **(A)** Fewer failures of blink suppression (reduced number of erroneous blinks during suppression). **(B)** Reduced activation in individualized postcentral gyrus target regions; brain image displays 5 mm-radius spheres centered around each individualized postcentral gyrus target coordinate for each participant (one color per participant). **(C)** Reduced activation in other key regions of the urge-network, i.e., insula and mid-cingulate cortices. Parameter estimates were extracted from the bilateral insula and mid-cingulate masks created from the automated anatomical labelling (AAL) in Pickatlas (Maldjian et al., 2003). **(D)** Greater reduction in self-reported urge to engage in OCD compulsions, as rated on the visual analogue scale (VAS). Ratings on the VAS were converted to a percentage score (refer to main text). Change scores on the VAS were calculated by subtracting VAS ratings taken immediately before (i.e., “pre”) and after (i.e., “post”) TMS (pre minus post) at both inhibitory postcentral gyrus TMS and sham TMS sessions.

Participants were accurate only 42% of the time when guessing which visit had delivered active or sham stimulation, indicating that blinding procedures were highly effective. Compared with sham stimulation, active inhibitory TMS delivered to the postcentral gyrus resulted in: (1) fewer failures of blink suppression (Figure 4A), (2) reduced activation in the individualized postcentral gyrus target region (Figure 4B), (3) reduced activation in other core urge network regions, i.e., the insula and mid-cingulate (Figure 4C); and (4) greater pre-to-post simulation reduction in self-reported urge to perform OCD-related compulsions (via the VAS) (Figure 4D) (also see Supplementary Table 3). Figure 4B shows the individualized postcentral gyrus areas selected for each of the four patients.

## 4 Discussion

This brief report presents a novel, individualized, and data-driven approach to identifying neural targets for inhibitory TMS aimed at reducing sensory-based urges in OCD. Compared to sham stimulation, inhibitory TMS delivered to an individualized target in the postcentral gyrus was associated with fewer blink suppression failures, reduced neural activation in urge network regions including the target postcentral gyrus as well as the insula and mid-cingulate cortices, and a greater reduction in self-reported urge to engage in OCD-related compulsions. While the sample size was small, the observed results yielded medium to large effect sizes, encouraging the utilization of this approach in future, appropriately-powered studies.

To our knowledge no prior study using TMS in OCD has investigated a postcentral gyrus target or used a data-driven approach to identify and target a neural region underlying urge suppression using both symptom-based and behavioral criteria as presented in Study 1. The present findings revealing effects of inhibiting postcentral gyrus on urge-related behavior and brain function is consistent with prior research showing that postcentral gyrus is involved in sensorimotor processing and is part of the neurocircuitry of sensory phenomena (Subirà et al., 2015; Brown et al., 2019; Shephard et al., 2021). However, the observed improvements may also be related to more widespread changes in additional urge network regions, including cingulate cortex and insula. Given that OCD is a heterogeneous condition, this study highlights the potential of using individualized, data-driven approaches to target hard-to-treat sensory phenomena in OCD.

There are several limitations in the current study. First, as with most TMS research, the current study did not account for individual differences in skull thickness and variation in scalp-to-cortex distance at different points of the head. As TMS intensity decays with increasing distance from the scalp, future studies may consider utilizing depth-corrected strategies to address individual variation in cortical distance to avoid under- or over-stimulation (Stokes et al., 2005). Second, Study 1 had an unbalanced sample, with significantly fewer healthy controls (*n* = 23) than individuals with OCD (*n* = 69). Additionally, the sample size for the TMS investigation (Study 2) is very small; however, this was intended to be a proof-of-concept study that can guide the development of larger, appropriately powered investigations. Future work is needed to validate the current study findings using larger samples for both Studies 1 and 2. Finally, this study chose a sham target distant from the active target in order to minimize participants directly comparing sensations between stimulation sessions, although this is not typical (Loo et al., 2000; Davis et al., 2013; Duecker and Sack, 2015). Nevertheless, results suggest that blinding procedures in the current study are still effective as participants were not successful in determining whether they received active or sham stimulation. Despite these limitations, our preliminary findings are encouraging and indicate that data-driven target selection based on a combination of behavioral and clinical factors may improve outcomes in neuromodulation studies in patients with psychiatric disorders. Overall, our study approach is novel and conceptually rigorous, building upon a strong foundation of prior research, focusing on the neurocircuit mechanisms of sensory-based urges. Future studies may build on this work, potentially leading to more precise and effective interventions.

## Data Availability

The raw data supporting the conclusions of this article will be made available by the authors, without undue reservation.
