# Supplementary material for: Personalized non-invasive neuromodulation for sensory-based urge suppression in individuals with OCD: a proof-of-concept investigation
Source: Front Hum Neurosci. 2025 Jun 24;19:1587644. doi: 10.3389/fnhum.2025.1587644 (PMC12234486; doi:10.3389/fnhum.2025.1587644)
Supplement: Supplementary file 1 [file Data_Sheet_1.pdf]

Personalized non-invasive neuromodulation for sensory-based urge suppression in individuals with OCD: a proof-of-concept investigation Supplement

Supplemental Information

1. Study 1 2

1.1 Participants..... 2

1.2 Inclusion/exclusion criteria..... 2

1.3 Data-cleaning for eyeblink data..... 2

1.4 Scales administered..... 2

1.5 Medication 2

1.6 Comorbid conditions..... 3

1.7 Neuroimaging data acquisition 3

1.7.1 Neuroimaging data preprocessing 3

1.8 Group differences in blinking behavior during the UFA fMRI task 4

2. Study 2 4

2.1 Participants..... 4

2.2 Medication 4

2.3 Comorbid conditions..... 4

Supplementary Table 1. Demographics, clinical, and behavioral information of full OCD sample (n=69) and healthy controls (n=23). 5

Supplementary Table 2. Demographics, clinical, behavioral information, and postcentral gyrus activation in OCD sub-sample (n=37) and healthy controls (n=23). 6

Supplementary Table 3. Demographics, clinical, behavioral information, and neural activation for OCD sample (n=4) in Study 2. 7

3. References..... 8

1. Study 1

1.1 Participants

A total of 69 patients with OCD and 23 controls completed the study between May 2017 and September 2020, of which, 11 patients and 2 controls were recruited and scanned at scanned at Icahn School of Medicine at Mount Sinai (ISMMS), 11 patients and 11 controls were recruited and scanned at Nathan Kline Institute for Psychiatric Research (NKI), and 47 patients and 10 controls were recruited at New York University Grossman School of Medicine (NYUSoM) and scanned at NKI. The study protocol was approved by the Institutional Review Boards at each institution and all subjects provided written informed consent. The dataset described in this paper is part of a larger set of neuroimaging studies, and overlapped a sample of 27 patients and 11 controls previously included in a published neuroimaging study (Stern et al., 2020).

1.2 Inclusion/exclusion criteria

All patients met DSM-5 criteria for OCD and were excluded for lifetime presence of bipolar disorder, psychotic disorder, or moderate/severe alcohol or substance use disorder.

Healthy controls were excluded for any lifetime presence of Axis I diagnoses. Diagnoses were made by a trained rater using the Mini International Neuropsychiatric Interview (MINI; (Sheehan et al., 1998)). All patients had OCD as their primary diagnosis as determined by the MINI, even if comorbid conditions were present.

1.3 Data-cleaning for eyeblink data

Eyeblinks during the task were measured via pupil occlusion using an Eyelink 1000-Plus device (SR Research, 2016) while fMRI data was acquired. The following guidelines were adhered:

1. Blinks occurring in the first second of each trial were excluded as they may have been due to an orienting response to the start of the trial;
2. Only device-designated blinks with durations between 50-2000-ms were included;
3. Participants were excluded from analyses if:
   i) More than 50% of their blinks were excluded (6 Patients; 2 Controls)
   ii) The average number of blinks during the 30s free-blinking blocks was less than either the first 30s or second 30s of the entire 60s-suppression period (3 Patients; 1 Control)
   iii) Their average number of blinks during free-blinking blocks was greater than 30 (3 Patients)
   iv) Other criteria were met:
      - General eye-tracker malfunction (e.g., could not calibrate successfully or no blinks were registered at all) (19 Patients; 10 Controls)
      - Participants fell asleep during the task (4 Patients)
      - Participants had their eyes closed during the task (1 Patient)

1.4 Scales administered

Overall OCD severity was measured using the total score from the clinician-administered Yale-Brown Obsessive Compulsive Scale (Y-BOCS), ranging from 0 (no symptoms) to 40 (severe symptoms) (Goodman et al., 1989). Sensory phenomena were measured using the total score from the clinician-administered University of Sao Paolo's Sensory Phenomena Scale, ranging from 0 (no sensory phenomena) to 15 (severe sensory phenomena) (Rosario et al., 2009; Sampaio et al., 2014).

1.5 Medication

Out of the total sample of 69 patients with OCD, 34 (49%) were currently not taking psychotropic medications; the remaining 35 patients were taking monoaminergic neurotransmission (serotonin reuptake inhibitor, serotonin modulator and stimulator, tricyclic antidepressants, n=32), atypical antipsychotics (n=4),

bupropion (n=3), topiramate (n=2), clonidine (n=1), and benzodiazepines (n=9). All patients refrained from taking benzodiazepines on the day of scanning. All control participants were free of current psychotropic medications, with the exception of one control, who was taking low-dose fluoxetine (below threshold for therapeutic effects) for post-menopausal hot flashes and gabapentin for neuralgia.

1.6 Comorbid conditions

Diagnoses were made according to The Diagnostic and Statistical Manual of Mental Disorders, Fifth Edition (DSM-5) by a trained rater using the Mini International Neuropsychiatric Interview (MINI) (Sheehan et al., 1998). All patients met criteria for OCD and were excluded for lifetime presence of bipolar disorder, psychotic disorder, or moderate/severe alcohol or substance use disorder. Out of the 69 patients, 21 (30%) did not have any current comorbid disorders; the remaining 48 (70%) had at least one current comorbid disorder, including generalized anxiety disorder (n=25), excoriation disorder (n=12), attention deficit hyperactivity disorder (n=11), panic disorder (n=12), body dysmorphic disorder (n=7), agoraphobia (n=8), social anxiety disorder (n=7), post-traumatic stress disorder (n=5), hoarding disorder (n=3), binge eating disorder (n=3), mild alcohol use disorder (n=3), trichotillomania (n=2), Tourette's disorder (n=2), mild substance use disorder (n=3), major depressive disorder (n=4), illness anxiety disorder (n=3), bulimia nervosa (n=3), somatic symptom disorder (n=2), and persistent tic disorder (n=2). All control participants were free of current or past diagnoses, with the exception of one participant, who had current alcohol use disorder (mild).

1.7 Neuroimaging data acquisition

All MRI scanning occurred on Siemens 3T scanners. ISMMS-recruited participants (11 patients and 2 controls) were scanned on a Siemens 3T MAGNETOM Skyra, and NYUSoM- and NKI-recruited participants (NYUSoM: 47 patients and 10 controls; NKI: 11 patients and 11 controls) were scanned on the 3T MAGNETOM TrioTim, with both sites using a 32-channel head coil, with sequences harmonized between the two scanning sites. Structural data was acquired using a T1-weighted MP-RAGE protocol at both ISMMS and NKI – Repetition time [TR]=2400ms, flip angle=8°, field-of-view [FOV]=256mm, 0.80mm isotropic voxels. The orientation of acquisition, echo time (TE), and number of slices were different between the two scanning sites (ISMMS: transverse acquisition, TE=2.07ms, 224 slices; NKI: sagittal acquisition, TE=2.01ms, 208 slices). Task fMRI was acquired using a high-resolution multiband-accelerated echo-planar sequence for full brain coverage (TR=1000ms, flip angle=60°, FOV=228mm, 72 slices, 2.1mm isotropic voxels without gap, acceleration factor=6, ascending interleaved order with anterior-to-posterior phase encoding). In order to match all other aspects of the sequences as closely as possible, the echo times (TEs) and acquisition orientation were slightly different between the two scanning sites (TE=25ms with oblique acquisition of T>C-20.0 at ISMMS, and TE=25.4ms with oblique acquisition of T>C-10.2 at NKI) (see below for description of post-hoc analyses accounting for residual neural differences between the sites). The first ten volumes were discarded to allow magnetization to reach equilibrium. The UFA-fMRI task was presented using E-Prime 2.0 software (Psychology Software Tools, 2016) and synchronized with scanning. Stimuli were back-projected in the scanner via a magnet-compatible projector, and participants viewed the stimuli from a mirror mounted above their head.

1.7.1 Neuroimaging data preprocessing

Preprocessing was performed using a combination of Statistical Parametric Mapping v.12 scripts taken from the Human Connectome Project preprocessing pipeline (Glasser et al., 2013; Glasser et al., 2016), AFNI (v.10.6, "3dSkullStrip"), and FSL v.5.0.10. Structural images were skull-stripped, nonlinearly corrected for gradient field distortion, and normalized to an MNI template (the "tissue probability map" [tpm] image in SPM v.12). Preprocessing for functional images included gradient nonlinearity distortion correction, realignment to the first volume of the run, normalization to MNI template, and spatial smoothing using a 6mm kernel. Six rigid-body realignment parameters (3 for translation: X, Y, and Z; and 3 for rotation: pitch, roll, yaw) were produced for each subject following the realignment step. Registrations of T1-weighted and

BOLD images to the MNI template were checked manually for each participant as part of our quality control procedures.

Following preprocessing, a fixed-effects model was created at the individual subject level using the general linear model approach to model the BOLD signal during the task as implemented in SPM v.12. Early suppression (first 30 seconds of 60-second suppression period) and late suppression (second 30 seconds of suppression period) were modelled as separate block regressors to allow for the differentiation of brain activation based on the build-up of the urge over time as we have previously done (Stern et al., 2020). Free-blinking blocks, blink-recovery periods, and rating periods were included in the model to account for variance. Six realignment parameters were also included as regressors-of-no-interest to further reduce variance associated with residual movement after realignment. Additional motion and artifact corrections were implemented with spike regression (Ciric et al., 2017) using the Artifact Detection Toolbox (Mazaika et al., 2005). Volumes with framewise displacement >2 mm in translation or >1° in rotation or global signal *Z*-value >9 were regressed out of the data by specifying them as regressors-of-no-interest in individual subject-level models.

At the group level, random-effects models compared brain activation differences between subgroups for each early and late suppression periods separately using two-sample *t*-tests. Despite sequence harmonization, all group-level imaging analyses specified scan site as a group-level covariate-of-no-interest to account for variance related to differences in image quality between the scanning sites, consistent with recommendations by McNeish and Kelley (McNeish and Kelley, 2019) and approaches used by multi-center studies (Glover et al., 2012; Forsyth et al., 2014).

1.8 Group differences in blinking behavior during the UFA fMRI task

As reported in our prior work (Stern et al., 2020; Bragdon et al., 2023; Eng et al., 2024), OCD patients showed more failures of blink suppression ($t(90)=2.78$, $p=0.007$) than control samples, but the groups did not differ during free-blinking ($p>0.05$) (Supplementary Table 1).The sample used in the present study overlaps with that reported in Stern et al., 2020, Bragdon et al., 2023, and Eng et al., 2024. As such, this does not represent an independent replication of the group difference finding.

2. Study 2

2.1 Participants

Supplementary Table 3 presents demographic, behavioral, and clinical characteristics of the four patients with OCD who completed Study 2.

2.2 Medication

Out of the four patients with OCD, two were (50%) were currently not taking psychotropic medications; the remaining two patients were taking alprazolam (n=1), bupropion (n=1), buspirone (n=1), and clonidine (n=1). The patient who was prescribed alprazolam refrained from taking it on the day of scanning.

2.3 Comorbid conditions

Following the same procedures in Study 1, diagnoses were made according to The Diagnostic and Statistical Manual of Mental Disorders, Fifth Edition (DSM-5) by a trained rater using the Mini International Neuropsychiatric Interview (MINI) (Sheehan et al., 1998). All four patients met criteria for at least one current comorbid disorder, including social anxiety disorder (n=3), generalized anxiety disorder (n=2), attention deficit hyperactivity disorder (n=2), persistent tic disorder (n=1), excoriation disorder (n=3), hoarding disorder (n=1), agoraphobia (n=2), body dysmorphic disorder (n=1), Tourette's disorder (n=1), and somatic symptom disorder (n=1).

Supplementary Table 1. Demographics, clinical, and behavioral information of full OCD sample (n=69) and healthy controls (n=23).

Notes. Early-suppression blink counts, late-suppression blink counts, free-blinking blink counts, and in-scanner urge intensity rating are average scores.
[a] Average blink counts were square-root transformed.
[b] Early-suppression refers to early phase of suppression (first 30 seconds of the 60-second suppression period; termed "suppression" in the main text); late-suppression refers to late phase of suppression (second 30 seconds of the 60-second suppression period).
Abbreviations: *Y-BOCS*, Yale-Brown Obsessive Compulsive Scale; *d*, Cohen's *d* for independent samples.
$^{**}P < 0.01$

	OCD (n=69)		Controls (n=23)		
	Mean	*SD*	Mean	*SD*	OCD Subgroup Comparisons
**Demographics**					
Age (years)	31.51	11.37	32.30	11.39	*n.s.*
Education (years)	15.64	2.04	16.26	1.86	*n.s.*
Sex assigned at birth (M:F [% F])	25:44 [63.8%]		8:15 [65.2%]		*n.s.*
**Clinical**					
Y-BOCS total	23.76	5.23	-	-	-
Sensory Phenomena Scale total	7.55	3.59	-	-	-
**UFA Task**					
Early-suppression blink counts[a,b]	1.65	0.86	1.09	0.80	OCD>Controls, $t(90)=2.78^{**}$, d=0.66
Late-suppression blink counts[a,b]	1.85	0.94	1.26	0.72	OCD>Controls, $t(90)=2.78^{**}$, d=0.66
Free-Blinking blink counts[a]	3.80	0.87	3.57	0.91	*n.s.*
In-scanner urge intensity rating	3.76	0.93	3.57	1.04	*n.s.*

Supplementary Table 2. Demographics, clinical, behavioral information, and postcentral gyrus activation in OCD sub-sample (n=37) and healthy controls (n=23).

Notes. This sub-sample of OCD patients (n=37) contributed to the neural activation analysis in comparison to controls (Study 1). Patients in the sub-sample were selected for exhibiting erroneous blinks greater than the median value of the full OCD sample during early suppression (i.e., >1.70). Information in this table is presented for descriptive purposes only.
Early-suppression blink counts, late-suppression blink counts, free-blinking blink counts, and in-scanner urge intensity rating are average scores.
[a] Average blink counts were square-root transformed.
[b] Early-suppression refers to early phase of suppression (first 30 seconds of the 60-second suppression period; termed “suppression” in the main text); late-suppression refers to late phase of suppression (second 30 seconds of the 60-second suppression period).
[c] Postcentral gyrus ROI is a 7mm-radius sphere centered on MNI coordinates 60 -26 42 derived from Study 1. Estimated marginal means of the parameter estimates are presented, corrected for the effect of scan site.
Abbreviations: *Y-BOCS*, Yale-Brown Obsessive Compulsive Scale.

	OCD (n=37)		Controls (n=23)	
	Mean	*SD*	Mean	*SD*
**Demographics**				
Age (years)	33.43	12.98	32.30	11.39
Sex assigned at birth (M:F [% F])	11:26 [70.3%]		8:15 [65.2%]	
**Clinical**				
Y-BOCS total	24.19	5.57	-	-
Sensory Phenomena Scale total	8.38	3.48	-	-
**UFA Task**				
Early-suppression blink counts[a,b]	2.30	0.53	1.09	0.80
Late-suppression blink counts[a,b]	2.50	0.64	1.26	0.72
Free-Blinking blink counts[a]	4.12	0.76	3.57	0.91
In-scanner urge intensity rating	3.99	0.89	3.57	1.04
**Neural Activation During Suppression**	Mean	*SE*	Mean	*SE*
Activation in postcentral gyrus ROI[c]	0.22	0.76	-0.34	0.09

Supplementary Table 3. Demographics, clinical, behavioral information, and neural activation for OCD sample (n=4) in Study 2.

Notes. All four patients with OCD completed Study 1.
Early-suppression blink counts, late-suppression blink counts, free-blinking blink counts, and in-scanner urge intensity rating are average scores.
[a] Average blink counts were square-root transformed.
[b] Early-suppression refers to early phase of suppression (first 30 seconds of the 60-second suppression period; termed "suppression" in the main text); late-suppression refers to late phase of suppression (second 30 seconds of the 60-second suppression period).
[c] Mean parameter estimates were extracted from 5mm-radius spheres centered on individualized coordinates in the postcentral gyrus, and bilateral masks of the insula and mid-cingulate cortices created from pickatlas toolbox.
[d] Visual analogue scale captured self-reported urge to engage in OCD compulsions, converted to a percentage score. Change score (*pre minus post*) was calculated by subtracting VAS rating taken immediately before (i.e., "pre") and after (i.e., "post") TMS.
Abbreviations: *Y-BOCS*, Yale-Brown Obsessive Compulsive Scale.

	OCD (*n*=4)				
	Mean	*SD*			
**Demographics**					
Age (years)	26.25	2.87			
Sex assigned at birth (M:F [% F])	0: 4 [100.0%]				
**Clinical**					
Y-BOCS total	19.13	2.66			
Sensory Phenomena Scale total	8.13	2.02			
	Sham TMS		Active inhibitory TMS		
	Mean	*SD*	Mean	*SD*	Hedge's *g*
**UFA Task**					
Early-suppression blink counts[a,b]	1.60	0.80	1.22	0.91	0.32
Late-suppression blink counts[a,b]	1.86	0.58	1.63	0.84	0.21
Free-blinking blink counts[a]	5.06	0.72	4.43	0.49	0.71
In-scanner urge intensity rating	3.03	0.81	2.94	0.55	0.09
**Neural Activation During Suppression**					
Activation in postcentral gyrus ROI[c]	0.37	0.33	0.01	0.48	0.62
Activation in bilateral insula ROI[c]	0.26	0.17	0.09	0.12	0.87
Activation in bilateral mid-cingulate ROI[c]	0.24	0.19	0.06	0.26	0.60
**VAS**					
Pre-Post TMS urge to engage in OCD compulsions (%)[d]	0.54	2.40	2.43	1.69	0.64

3. References

Bragdon, L.B., Nota, J.A., Eng, G.K., Recchia, N., Kravets, P., Collins, K.A., et al. (2023). Failures of Urge Suppression in Obsessive-Compulsive Disorder: Behavioral Modeling Using a Blink Suppression Task. *J Obsessive Compuls Relat Disord* 38**,** 100824. doi: 10.1016/j.jocrd.2023.100824.

Ciric, R., Wolf, D.H., Power, J.D., Roalf, D.R., Baum, G.L., Ruparel, K., et al. (2017). Benchmarking of participant-level confound regression strategies for the control of motion artifact in studies of functional connectivity. *Neuroimage* 154**,** 174-187.

Eng, G.K., De Nadai, A.S., Collins, K.A., Recchia, N., Tobe, R.H., Bragdon, L.B., et al. (2024). Identifying subgroups of urge suppression in Obsessive-Compulsive Disorder using machine learning. *Journal of Psychiatric Research* 177**,** 129-139.

Forsyth, J.K., McEwen, S.C., Gee, D.G., Bearden, C.E., Addington, J., Goodyear, B., et al. (2014). Reliability of functional magnetic resonance imaging activation during working memory in a multi-site study: analysis from the North American Prodrome Longitudinal Study. *Neuroimage* 97**,** 41-52. doi: 10.1016/j.neuroimage.2014.04.027.

Glasser, M.F., Smith, S.M., Marcus, D.S., Andersson, J.L.R., Auerbach, E.J., Behrens, T.E.J., et al. (2016). The human connectome project's neuroimaging approach. *Nature neuroscience* 19(9)**,** 1175-1187.

Glasser, M.F., Sotiropoulos, S.N., Wilson, J.A., Coalson, T.S., Fischl, B., Andersson, J.L., et al. (2013). The minimal preprocessing pipelines for the Human Connectome Project. *NeuroImage* 80**,** 105-124. doi: https://doi.org/10.1016/j.neuroimage.2013.04.127.

Glover, G.H., Mueller, B.A., Turner, J.A., van Erp, T.G.M., Liu, T.T., Greve, D.N., et al. (2012). Function biomedical informatics research network recommendations for prospective multicenter functional MRI studies. *Journal of Magnetic Resonance Imaging* 36(1)**,** 39-54. doi: https://doi.org/10.1002/jmri.23572.

Goodman, W.K., Price, L.H., Rasmussen, S.A., Mazure, C., Fleischmann, R.L., Hill, C.L., et al. (1989). The Yale-Brown Obsessive Compulsive Scale. I. Development, use, and reliability. *Archives of General Psychiatry* 46**,** 1006-1011.

Mazaika, P.K., Whitfield, S., and Cooper, J.C. (2005). Detection and repair of transient artifacts in fMRI data. *Neuroimage* 26(Suppl 1)**,** S36.

McNeish, D., and Kelley, K. (2019). Fixed effects models versus mixed effects models for clustered data: Reviewing the approaches, disentangling the differences, and making recommendations. *Psychological Methods* 24(1)**,** 20-35. doi: 10.1037/met0000182.

Psychology Software Tools, I. (2016). "E-Prime 3.0".).

Rosario, M.C., Prado, H.S., Borcato, S., Diniz, J.B., Shavitt, R.G., Hounie, A.G., et al. (2009). Validation of the University of Sao Paulo Sensory Phenomena Scale: initial psychometric properties. *CNS Spectr* 14(6)**,** 315-323. doi: 10.1017/s1092852900020319.

Sampaio, A.S., McCarthy, K.D., Mancuso, E., Stewart, S.E., and Geller, D.A. (2014). Validation of the University of São Paulo's Sensory Phenomena Scale -- English version. *Compr Psychiatry* 55(5)**,** 1330-1336. doi: 10.1016/j.comppsych.2014.02.008.

Sheehan, D.V., Lecrubier, Y., Sheehan, K.H., Amorim, P., Janavs, J., Weiller, E., et al. (1998). The Mini-International Neuropsychiatric Interview (MINI): the development and validation of a structured diagnostic psychiatric interview for DSM-IV and ICD-10. *Journal of Clinical Psychiatry* 59(20)**,** 22-33.

SR Research (2016). "Eyelink 1000 plus".).

Stern, E.R., Brown, C., Ludlow, M., Shahab, R., Collins, K., Lieval, A., et al. (2020). The buildup of an urge in obsessive–compulsive disorder: Behavioral and neuroimaging correlates. *Human Brain Mapping* 41(6)**,** 1611-1625. doi: 10.1002/hbm.24898.
